# A new fossil mantis shrimp and the convergent evolution of a lobster-like morphotype

**DOI:** 10.7717/peerj.11124

**Published:** 2021-04-16

**Authors:** Carolin Haug, Joachim T. Haug

**Affiliations:** 1 Biology II, Ludwig-Maximilians-Universität München, Planegg-Martinsried, Germany; 2 GeoBio-Center, Ludwig-Maximilians-Universität München, Munich, Germany

**Keywords:** Tyrannosculda laurae, Stomatopoda, Stomatoreptantia, Isopoda, Reptantia, Convergence, Upper Jurassic, Solnhofen limestones

## Abstract

Eumalacostracan crustaceans all have a more or less stereotypic body organisation in the sense of tagmosis. Originally, this included a head with six segments (ocular segment plus five appendage-bearing segments), a thorax region with eight segments, and a pleon with six segments. Interestingly, despite these restrictions in variability in terms of tagmosis, the morphological diversity within Eumalacostraca is rather high. A group providing representative examples that are commonly known is Decapoda. Decapodan crustaceans include shrimp-like forms, lobster-like forms and crab-like forms. The stem species of Eucarida, the group including Decapoda and Euphausiacea, presumably possessed a rather shrimp-like morphology, quite similar to the stem species of Eumalacostraca. Also two other lineages within Eumalacostraca, namely Hoplocarida (with the mantis shrimps as modern representatives) and Neocarida (with the sister groups Thermosbaenacea and Peracarida) evolved from the shrimp-like body organisation to include a lobster-like one. In this study, we demonstrate that the stepwise evolution towards a lobster morphotype occurred to a certain extent in similar order in these three lineages, Hoplocarida, Eucarida and Peracarida, leading to similar types of derived body organisation. This evolutionary reconstruction is based not only on observations of modern fauna, but especially on exceptionally preserved Mesozoic fossils, including the description of a new species of mantis shrimps bridging the morphological gap between the more ancestral-appearing Carboniferous forms and the more modern-appearing Jurassic forms. With this, Mesozoic eumalacostracans represent an important (if not unique) ‘experimental set-up’ for research on factors leading to convergent evolution, the understanding of which is still one of the puzzling challenges of modern evolutionary theory.

## Introduction

Mantis shrimps (Stomatopoda) are rather distinct representatives of Malacostraca (‘higher crustaceans’) and can hence be easily recognised via their numerous unique traits such as highly specialised eyes (e.g. Marshall et al., 1991a, 1991b; Marshall, Cronin & Kleinlogel, 2007; Daly et al., 2016) and powerful raptorial apparatus (e.g. Patek & Caldwell, 2005; Haug et al., 2010; Claverie, Chan & Patek, 2011). Nowadays, Hoplocarida, the group including modern mantis shrimps and their fossil relatives, is recognised as an ingroup of Eumalacostraca (e.g. Richter & Scholtz, 2001; Schwentner et al., 2018). It is generally understood as the sister group to Caridoida, the group of malacostracans with a tail-flip response behaviour or caridoid escape reaction (including Decapoda, Euphausiacea, Syncarida and Neocarida, the latter including Thermosbaenacea and Peracarida; Richter & Scholtz, 2001). In former times, mantis shrimps have been hypothesised to be more closely related to different caridoid ingroups. Most interestingly, they have been closely allied to Decapoda (e.g. Schram, 1986). This was most likely based on the fact that mantis shrimps share numerous characters with the latter. These similarities can make the distinction of fossil representatives of Decapoda and those of Hoplocarida quite difficult (e.g. Schram, 1986). Yet, due to their relatively distant phylogenetic relationships and the absence of hoplocaridan characters in other eumalacostracans, all these similarities between Decapoda and Hoplocarida are best interpreted as convergently evolved. Thus, the comparison of the evolutionary lineages of the two groups is an ideal case to study potential convergent evolutionary patterns.

Decapoda is represented by quite different morphotypes in the modern fauna. Besides the prominent ‘crab’ morphotype in representatives of Meiura, two further types are generally differentiated (e.g. Glaessner, 1969; Förster, 1985): the more plesiomorphic ‘shrimp’ morphotype in representatives of Dendrobranchiata, Stenopodidea, and Caridea, and the more derived ‘lobster’ morphotype in non-crab type representatives of Reptantia, previously referred to as Macrura. The evolutionary transition from a lobster morphotype to a crab morphotype has usually been addressed as ‘carcinisation’ or ‘brachyurisation’ (e.g. Števčić, 1971; McLaughlin & Lemaitre, 1997; McLaughlin, Lemaitre & Tudge, 2004; Ahyong, Schnabel & Macpherson, 2011; Scholtz, 2014; Keiler, Wirkner & Richter, 2017, and references therein). Consequently, one could call the transition from shrimp morphotype to lobster morphotype ‘reptantisation’. The latter transition has usually been viewed as an adaptive transition from swimming to crawling.

When comparing modern mantis shrimps (Verunipeltata; Haug et al., 2010) to decapodans, they appear in many aspects best comparable to lobster-like representatives of Decapoda. No real shrimp morphotype can be found among modern representatives of Stomatopoda (concerning adults, larvae are often very shrimp-like; e.g. Giesbrecht, 1910; Ahyong, Haug & Haug, 2014), yet the stem species of Eumalacostraca appears to have been shrimp-like. In consequence, modern mantis shrimps should have also evolved by a process of reptantisation, convergently to decapodan lobsters.

The comparison of two evolutionary lineages that might have evolved similar traits convergently, as possibly in the here discussed case, involves two aspects. First, it is interesting to see whether in both lineages the independent evolutionary ‘pathways’ of acquiring novelties appear to follow the same intrinsic restrictions. This can be tested by comparing the exact sequences of events in which the adaptations evolved, i.e. does step A always evolve before B, or can it evolve also in the reversed pattern? Identifying cases of such intrinsic restrictions give hints for certain adaptations being pre-requisites for others. Second to be compared are extrinsic factors. This relates to adaptations to specific selective pressures, i.e. do different lineages ‘respond’ in the same way to, for example, habitat changes? For such questions, seemingly convergently evolved lineages are a perfect historical type of ‘experimental setup’.

As pointed out, modern (adult) mantis shrimps do not represent the shrimp morphotype, but appear to have evolved from such a morphotype. Therefore, especially fossil representatives are of central interest for the here presented approach. As earlier studies have shown, the fossil record of mantis shrimps provides crucial insights into the early evolutionary history of Stomatopoda (e.g. Schram, 2007; Haug et al., 2010). Yet, in most former studies other aspects than the general body organisation have been in focus, e.g. the evolution of the raptorial apparatus.

We describe a new mantis shrimp species based on fossil specimens from the Upper Jurassic (about 150 million years old) of Southern Germany. Based on these data, we discuss the evolutionary process of reptantisation in mantis shrimps and compare in how far this process of morphotype evolution can be compared with similar processes in the evolutionary lineages of Decapoda and possibly other lineages within Eucrustacea.

## Materials and Methods

### Material

Specimens used in this study come from the collections of the Staatliches Museum für Naturkunde Stuttgart with repository numbers SMNS 67505 (here 017), SMNS 67592/1 (069; originally collection of Hermann Polz, Geisenheim), SMNS 67592/2 (070; originally collection of Hermann Polz, Geisenheim), SMNS 67593 (083; originally collection of Norbert Winkler, Stahnsdorf), and SMNS 67633 (090; originally collection of Michael Fecke, Lippstadt). Further material used in this study comes from the collections of Michael Fecke, Lippstadt (007), of Roger Frattigiani, Laichingen (013), of Norbert Winkler, Stahnsdorf (030), of Marina and Matthias Wulf, Rödelsee (037), and of Markus Gebert, Mainbernheim (064, 097).

### Documentation methods

Specimens were investigated and documented with a Zeiss Axioscope 2 epifluorescence compound microscope equipped with an Axiocam. Illumination with different wavelengths significantly enhances the contrast between the fossil exhibiting autofluorescence and the surrounding limestone matrix (e.g. Polz, 1972; Tischlinger, 2002, 2015; Haug, Haug & Ehrlich, 2008; Haug et al., 2009, 2011). Each image detail was recorded as a stack of images, which was then fused with the programmes CombineZM/ZP to overcome the limited depth of field. Limitations in the field of view were overcome by stitching fused images of adjacent image details until the entire specimen was documented (e.g., Haug, Haug & Ehrlich, 2008; Haug et al., 2009; Kerp & Bomfleur, 2011).

### Presentation methods

3D models of the new species (see below) and comparative material were reconstructed in the free software Blender. Drawings were performed in Adobe Illustrator CS2.

### Description

The description of morphological details follows the descriptive matrix approach (see Haug, Briggs & Haug (2012a) for details).

### Taxonomy

The electronic version of this article in Portable Document Format (PDF) will represent a published work according to the International Commission on Zoological Nomenclature (ICZN), and hence the new names contained in the electronic version are effectively published under that Code from the electronic edition alone. This published work and the nomenclatural acts it contains have been registered in ZooBank, the online registration system for the ICZN. The ZooBank LSIDs (Life Science Identifiers) can be resolved and the associated information viewed through any standard web browser by appending the LSID to the prefix http://zoobank.org/. The LSID for this publication is: urn:lsid:zoobank.org:pub:66FB761B-8C3E-4B57-8E0C-1055B2E8BD73. The online version of this work is archived and available from the following digital repositories: PeerJ, PubMed Central and CLOCKSS.

## Results

### Ontogeny

There are observable differences between the specimens. Yet, most of these relate to preservation. When considering these differences, there remains no character that would allow to differentiate two species within the material. We therefore must assume that these are conspecific.

**Figure 1:**
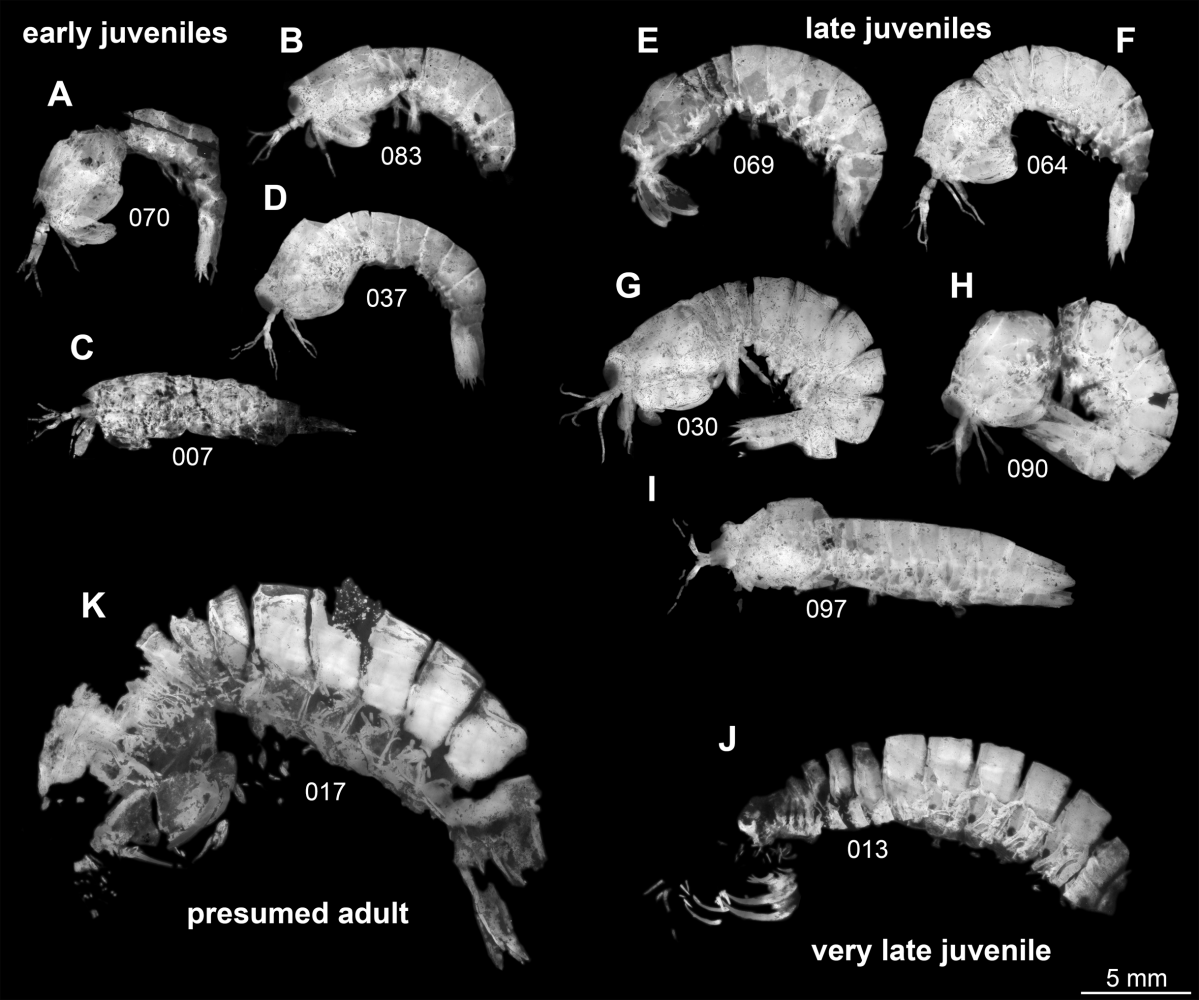
Overview over studied material of *Tyrannosculda laurae* n. gen. n. sp., roughly sorted by presumed ontogenetic phase. (A–D) Early juveniles. (E–I) Late juveniles. (J) Very late juvenile. (K) Presumed adult. The following specimens have been horizontally flipped to have their anterior end to the left side for a better comparability: 017, 030, 064, 069, 070, 083, 090. Figured in earlier publications: (C) Haug et al. (2010, their Fig. 2, no. 2); (D) Haug et al. (2010, their Fig. 2, no. 7); (G) Haug et al. (2010, their Fig. 2, no. 1); (J) Haug et al. (2010, their Fig. 2, no. 9); (K) Haug et al. (2009, their Fig. 3), Haug et al. (2010, their Fig. 2, no. 3), and Haug et al. (2012b, their Fig. 7A). Haug et al. (2009) was published under CC BY-NC-SA 4.0 licence, Haug et al. (2010) was published under CC BY 2.0 licence, and Haug et al. (2012b) was published under CC BY 4.0 licence.

Following this interpretation, the specimens investigated in this study appear to represent different ontogenetic stages of one species, from early juveniles to the supposed adult stage (Fig. 1). However, the material is not yet sufficient to reconstruct a complete sequence for this species. Also a statistical morphometric differentiation of different moult stages was not possible due to the limited amount of material.

### Morphological description

**Figure 2:**
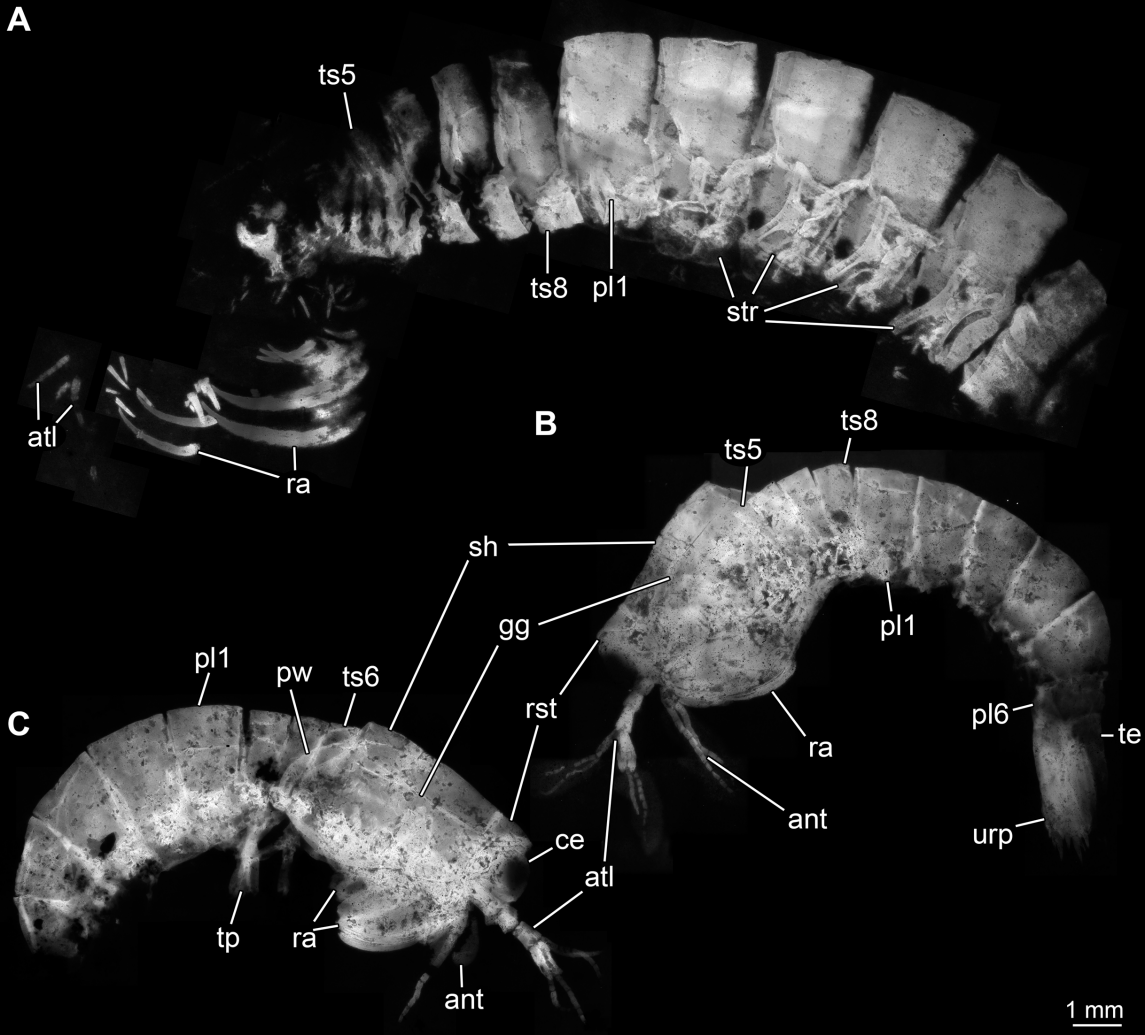
Habitus of *Tyrannosculda laurae* n. gen. n. sp., different ontogenetic stages. (A) Very late juvenile (adult-like morphology; specimen 013). (B and C) Earlier juveniles. (B) Later early juvenile (specimen 037). (C) Earlier juvenile (specimen 083) than in B. Abbreviations: ant, antenna; atl, antennula; ce, compound eye; gg, gastric groove; pl1–6, pleon segments 1–6; pw, posterior wing of shield; ra, raptorial appendage; rst, rostrum; sh, shield; str, sternite; te, telson; tp, thoracopod; ts5–8, trunk segments 5–8; urp, uropod.

Body organised into (presumably) 20 segments, ocular segment and 19 post-ocular appendage bearing segments (Figs. 2A and 2B). Dorsal area of anterior body forming prominent shield; unclear which segments contribute to this structure due to preservation. Shield roughly trapezoidal in lateral view (Fig. 2B). Anteriorly, small triangular rostral plate of about a quarter of the length of the main shield (in anterior-posterior dimension) jointed against shield (Figs. 2B and 2C). Shield laterally drawn out to envelop large parts of the anterior body (until about thorax segment 5); reaching further ventrally towards the posterior end, posteriorly almost concealing entire body height. Anteriorly, height of shield about half of its length; posteriorly, about three quarters. Postero-lateral edges of the shield drawn out into rounded wings, overhanging succeeding segments (Fig. 2C). Pronounced grooves (presumed gastric grooves) running from anterior to posterior at about two fifth of the height of the shield (measured anteriorly) (Figs. 2B and 2C).

Details of dorsal areas of ocular segment and (presumably) post-ocular segments 1–6 (antennular, antennal, mandibular, maxillulary, maxillary, first maxillipedal segment) unclear due to preservation.

Dorsal area of post-ocular segment 7 (second maxillipedal segment; trunk segment 2) weakly sclerotised, forming a narrow tergite (Fig. 2A). Reaching down far laterally (continuing into sternite?). Sclerite not in straight dorsal-ventral orientation, but tilted ventrally towards anterior by about 45°.

Dorsal area of post-ocular segment 8 (third maxillipedal segment; trunk segment 3) weakly sclerotised, forming a narrow tergite, slightly larger than preceding one (Fig. 2A). Reaching down far laterally (continuing into sternite?). Sclerite not in straight dorsal-ventral orientation, but tilted ventrally towards anterior by about 40°.

Dorsal area of post-ocular segment 9 (fourth maxillipedal segment; trunk segment 4) weakly sclerotised, forming a narrow tergite, slightly larger than preceding one (Fig. 2A). Reaching down far laterally (continuing into sternite?). Sclerite not in straight dorsal-ventral orientation, but tilted ventrally towards anterior by about 35°.

Dorsal area of post-ocular segment 10 (fifth maxillipedal segment; trunk segment 5) weakly sclerotised, forming a narrow tergite, slightly larger than preceding one (Fig. 2A). Reaching down far laterally (continuing into sternite?). Sclerite not in straight dorsal-ventral orientation, but tilted ventrally towards anterior by about 30°.

Dorsal areas of post-ocular segments 11–13 (trunk segments 6–8; thorax segments 6–8) each with a distinct tergite (Figs. 2B and 2C). Each tergite about one fifth of the length of the head shield. Reaching down far laterally, about two thirds of entire body height.

Dorsal areas of post-ocular segments 14–16 (trunk segments 9–11; pleon segments 1–3) each with a distinct tergite (Fig. 2). Each tergite about two fifths of the length of the head shield. Reaching down far laterally, about four fifths of entire body height.

Dorsal areas of post-ocular segments 17–19 (trunk segments 12–14; pleon segments 4–6) each with a distinct tergite (Fig. 2). Tergite of post-ocular segment 17 differing ontogenetically. In early juveniles, dorsal dimension similar to that of preceding tergite; ventral dimension shorter, overall height smaller, tapering distally. In later juveniles and presumed adults, tergite about two fifths of the length of the head shield; overall similar in dimensions to preceding tergite; reaching down far laterally, about four fifths of entire body height.

Tergite of post-ocular segment 18 differing ontogenetically. In early juveniles, medio-posteriorly armed with a pair of stout spines; dorsal dimension shorter than that of preceding tergite, ventral dimension even shorter, overall height even smaller, tapering distally. In later juveniles, dorsal dimension similar to that of preceding tergite; ventral dimension shorter, overall height smaller, tapering distally. In adults, tergite about two fifths of the length of the head shield; overall similar in dimensions to preceding tergite; reaching down far laterally, about four fifths of entire body height.

Tergite of post-ocular segment 19 in dorsal dimension shorter than preceding tergite; ventral dimension even shorter, overall height smaller, tapering distally.

Details of telson largely unknown due to preservation; preserved part appears elongate triangular.

Compound eyes arising from ocular segment; large, prominent, apparently with a short stalk (Fig. 1). Further details unknown due to preservation.

**Figure 3:**
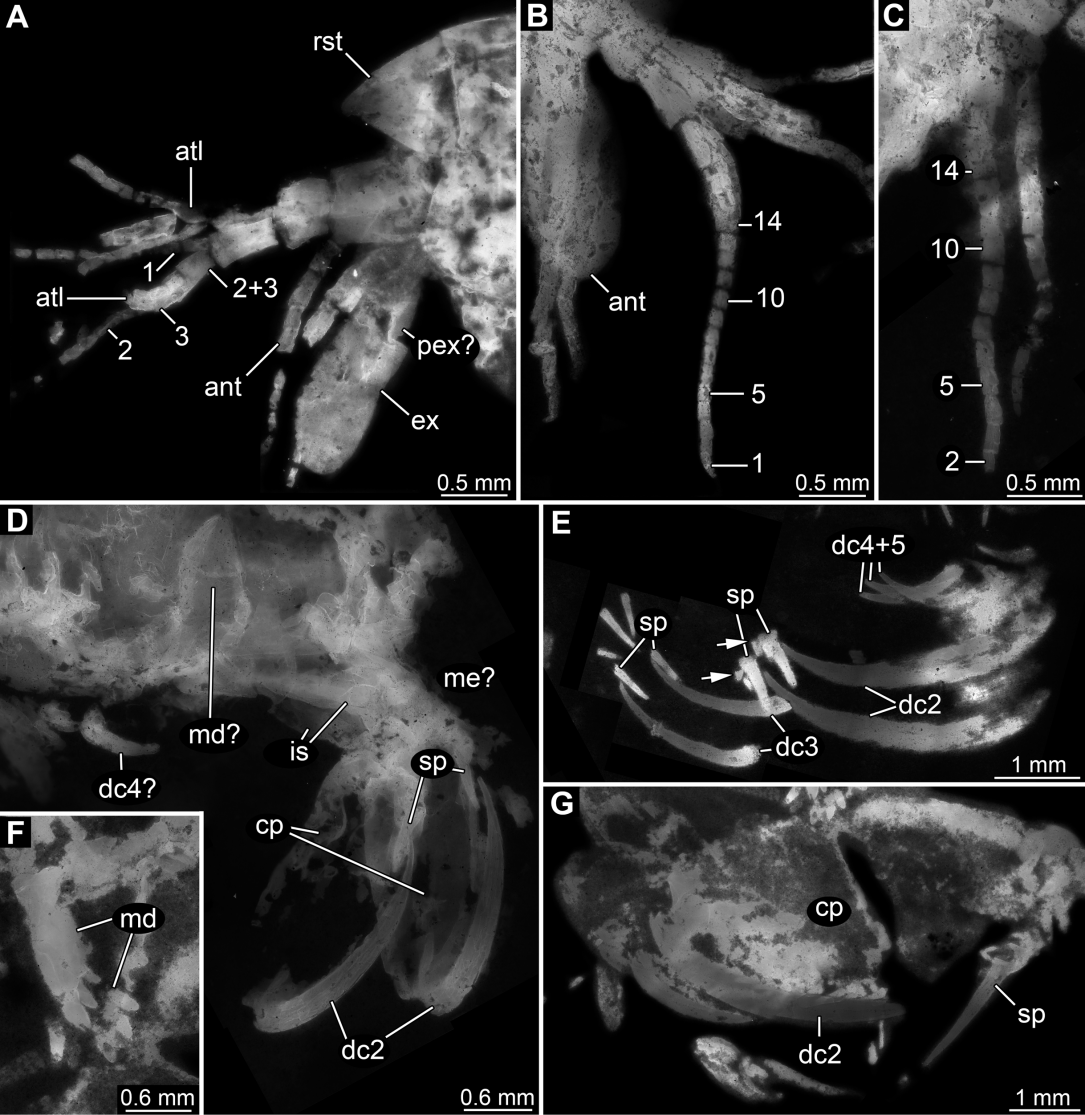
Details of anterior region of *Tyrannosculda laurae* n. gen. n. sp. (A–C) Close-up on antennula and antenna. (A) Specimen 007. (B) Specimen 030. (C) Specimen 064. (D and E) Close-up on raptorial appendages. (D) Specimen 069. (E) Specimen 013; arrows point to flanking spines. (F and G) Specimen 017. (F) Close-up on mandibles. (G) Close-up on distal region of major raptorial appendage. Abbreviations: 1–3, flagella of antennula; 2+3, element 4 of antennula of which flagella 2+3 arise; 1–14, flagellomeres of antennula; ant, antenna; atl, antennula; cp, carpo-propodus; dc2–5, dactylus of maxillipeds 2–5; ex, exopod; is, ischium; md, mandible; me?, presumed merus; pex?, possible proximal part of exopod; pp, propodus; ra, raptorial appendage; rst, rostrum; sp, spine. Figured in earlier publications: (A) Haug et al. (2010, their Fig. 3A) and Haug & Haug (2011, their Abb. 2B); (B) Haug et al. (2010, their Fig. 3A) and Haug & Haug (2011, their Abb. 2A); (E) Haug et al. (2010, their Fig. 5B) and Haug & Haug (2011, their Abb. 3C); (F and G) Haug et al. (2010, their Fig. 3C) and Haug & Haug (2011, their Abb. 2C). Haug et al. (2010) was published under CC BY 2.0 licence.

Appendage of post-ocular segment 1 (antennula) with four distinct elements and three distal multi-annulated flagella (Figs. 3A–3C). All four elements cylindrical, about twice as long (in proximal-distal axis) as wide (in diameter). Elements 1–3 about the same size, element 4 smaller. Flagellum 1 arising from element 3; with up to 14 flagellomeres; flagellomeres about as long as wide; distal flagellomeres tapering. Flagella 2 and 3 arising from element 4 (Fig. 3A).

Appendage of post-ocular segment 2 (antenna) with details of proximal region unclear (due to preservation), distally with endopod and exopod (Figs. 3A and 3B). Proximal region of endopod unclear (due to preservation), distally forming flagellum with at least 12 flagellomeres. Flagellomeres about as long as wide; distal flagellomeres tapering. Exopod paddle-shaped, longer than wide, about 3 times; possibly subdivided by a joint into a proximal and a distal region (Fig. 3A).

Appendage of post-ocular segment 3 (mandible) known from proximal region (coxal body; Figs. 3D and 3F). Elongate triangular with four finger-like enditic protrusions medially. No distal part (palpus) recognisable. It remains unclear whether this is the original condition or a preservational artefact.

Appendages of post-ocular segments 4–6 (maxillula, maxilla, first maxilliped) unknown due to preservation.

Appendage of post-ocular segment 7 (second maxilliped; large raptorial appendage) with details of proximal region unclear (due to preservation), distally with four distinct elements (in the following numbered from proximally to distally). Overall arrangement in a z-shape: distal three elements folded against proximal one; terminal element folded against sub-terminal one (Figs. 3D and 3G). Element 1 (ischium) cylindrical, elongate, at least 2.5 times as long as wide. Element 2 (merus) short, roughly triangular; exact outline unclear due to preservation. Element 3 (carpo-propodus; see Haug et al. (2016) for element determination of maxillipeds) massive, twice as wide as element 1, twice as long as wide. Rounded medially and laterally, widest in the middle. Median edge with indication of serrations or small spines in the very mature forms (absent in smaller forms, unclear if ontogenetic or preservational effect). Proximo-medially with a distinct jointed spine (Figs. 3E and 3G), about one quarter to one third of the length of element 2; straight, tapering distally; with two smaller flanking spines close to its base. Element 4 (dactylus) scimitar-shaped, curved “inwards” (Figs. 3D, 3E and 3G); slightly shorter than preceding element. Proximally significantly narrower than carpo-propodus, about one third of maximum width, tapering distally.

Appendage of post-ocular segment 8 (third maxilliped; raptorial appendage) with details of proximal region unclear (due to preservation), distally with two distinct elements. Element 1 (carpo-propodus) largely unknown due to preservation, with three distinct jointed spines medially, all prominent, straight, tapering distally (Fig. 3E). Jointed spine 1 far proximally; jointed spine 2 further distally, about half the size of spine 1; jointed spine 3 even further distally, only slightly smaller than spine 1. Element 2 (dactylus) scimitar-shaped, curved ‘inwards’; about half the size of dactylus of post-ocular appendage 7 (Fig. 3E).

Appendages of post-ocular segments 9 and 10 (fourth and fifth maxilliped; raptorial appendages) with details of proximal region unclear (due to preservation), distally with a single distinct element. This element 1 (dactylus) scimitar-shaped, curved ‘inwards’; slightly less than one third of dactylus of post-ocular appendage 7 (Fig. 3E).

**Figure 4:**
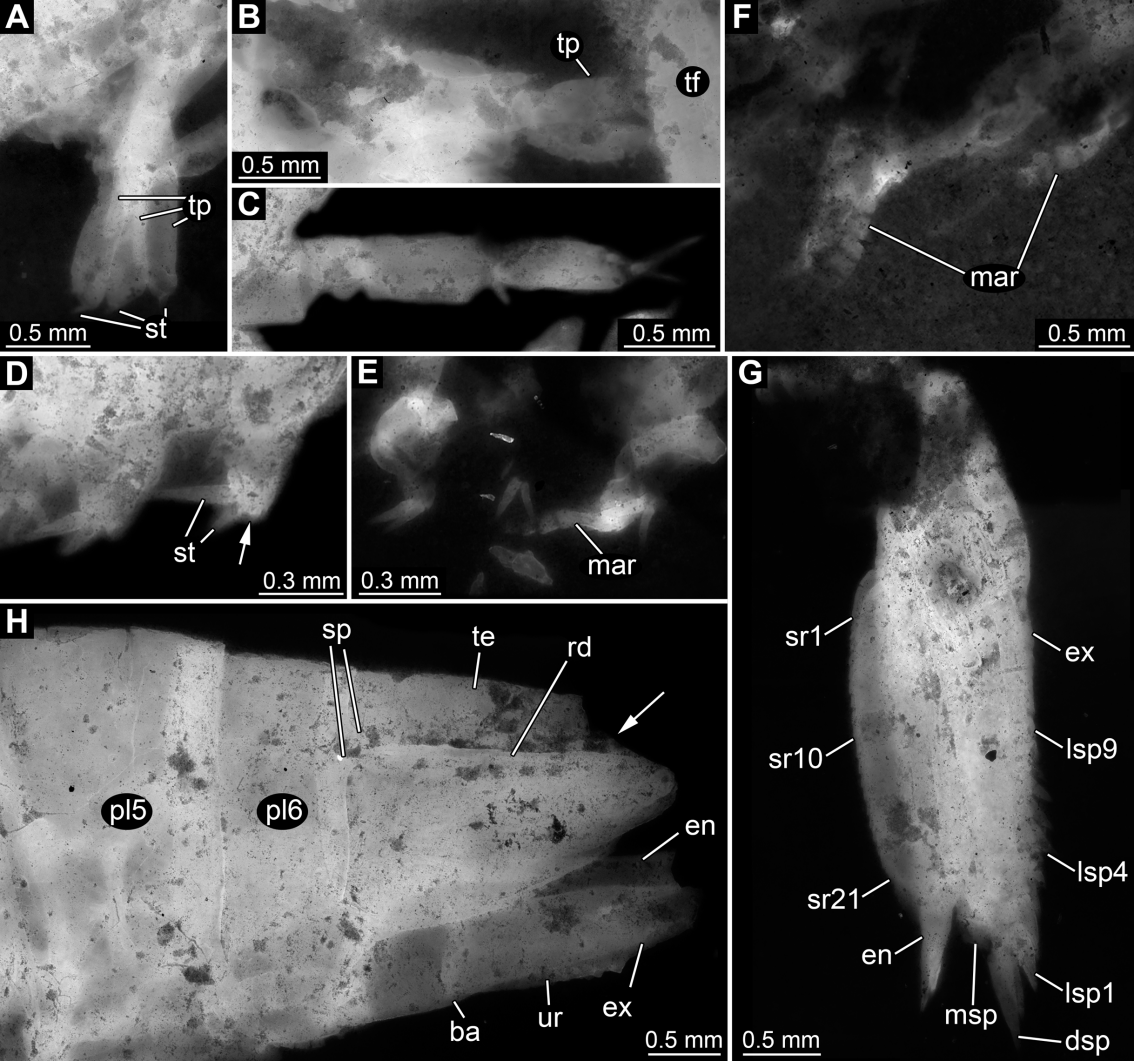
Details of posterior region of *Tyrannosculda laurae* n. gen. n. sp. (A–C) Close-up on walking appendages. (A) Specimen 083. (B) Specimen 090. (C) Specimen 030. (D–F) Close-up on pleopods. (D) Specimen 030; arrow points to possible third seta. (E) Specimen 064. (F) Specimen 070. (G) Close-up on uropod; specimen 064. (H) Posterior trunk end with tail fan; arrow points to broken off part of telson; specimen 097. Abbreviations: ba, basipod; dsp, distal spine; en, endopod; ex, exopod; lsp1–9, lateral spine 1–9; mar, multi-annulated ramus; msp, medio-distal spine; pl5–6, pleon segment 5–6; rd, ridge on telson; sp, spine; sr1–21, serration 1–21; st, seta; te, telson; tf, tail fan; tp, thoracopod; ur, uropod. Figured in earlier publications: (C) Haug et al. (2010, their Fig. 5C, D) and Haug & Haug (2011, their Abb. 2E). Haug et al. (2010) was published under CC BY 2.0 licence.

Appendages of post-ocular segments 11–13 (sixth to eighth thoracopod; walking appendages) overall tubular, with four distinct elements (Figs. 4A–4C). Element 1 (most proximal one) robust, comparably broad, exact length unclear due to preservation. Element 2 less massive than preceding element; about as long as broad. Element 3 about as broad as preceding element, but about 2.5 times as long; distally armed with a single seta. Element 4 (terminal element) slightly less broad than preceding element, about as long as preceding element; distally tapering to a rounded tip; tip armed with two setae.

Appendages of post-ocular segments 14–18 (first to fifth pleopod) with an inferred basipod (not directly observable) and two distal rami (unclear which one is the endopod and which one is the exopod). One ramus simple paddle-shaped, with two or three setae distally (Fig. 4D). Other ramus elongate, multi-annulated with four or five annuli (Figs. 4E and 4F); distally with a long seta.

Appendage of post-ocular segment 19 (uropod, sixth pleopod) with basipod carrying endopod and exopod distally (Fig. 4H). Basipod more or less rectangular, slightly broader than long. Endopod arising medio-distally from basipod; lanceolate to paddle-shaped, longer than wide, about 3 times. Distally drawn out into a prominent spine (Fig. 4G). Median edge armed with about 20 distinct distally pointing serrations (Fig. 4G). Exopod arising latero-distally from basipod; lanceolate to paddle-shaped, longer than wide, about 3 times. Distally drawn out into a prominent spine (Fig. 4G). Medio-distally with another prominent spine. Lateral edge with up to nine prominent movable spines (spine-like setae), decreasing in length towards the proximal end of the exopod (Fig. 4G).

### Description of new species

Eucrustacea sensu Walossek, 1999

Malacostraca Latreille, 1802

Eumalacostraca Grobben, 1892

Hoplocarida Calman, 1904

Stomatopoda Latreille, 1825

Unipeltata s. l. sensu Haug et al., 2010

*Tyrannosculda* n. gen.

Life Science Identifier: urn:lsid:zoobank.org:act:912A8E58-2C1E-4107-A40A-E693CBB121F7

Derivation of the name: Similarities to the raptorial apparatus of the Carboniferous species of *Tyrannophontes* and otherwise resemblance of *Sculda*.

Type species: *Tyrannosculda laurae* n. sp.

Life Science Identifier: urn:lsid:zoobank.org:act:27B4B4E9-C598-4140-B34C-267EBAD68F6B

Diagnosis: as for the species

Remarks: Although the specimens now assigned to the single species of *Tyrannosculda* were at first assigned to a species of *Sculda*, new details of their morphology exhibit significant differences (see details below).

**Figure 5:**
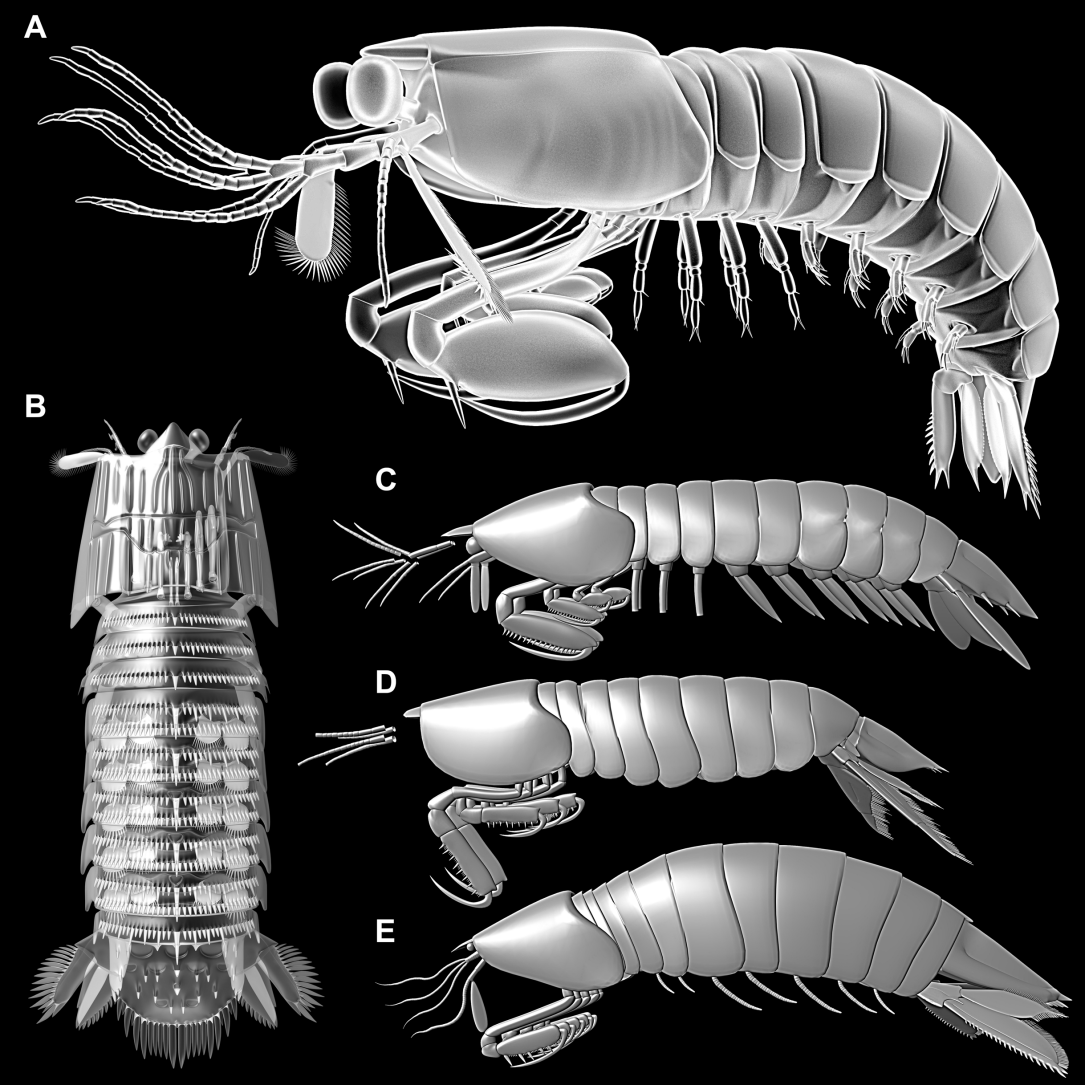
3D reconstructions of *Tyrannosculda laurae* and other fossil representatives of Stomatopoda. (A) *Tyrannosculda laurae*. (B) ?*Sculda pennata/spinosa*. (C) *Tyrannophontes theridion*. (D) *Gorgonophontes fraiponti*. (E) *Daidal schoellmanni*.

*Tyrannosculda laurae* n. gen. n. sp.

v 2009 *Sculda pennata* – Haug et al., pp. 2, 5, 9; table 1; fig. 3.

v 2010 ?*Sculda pusilla* – Haug et al., pp. 2, 3, 5, 6, 10, 12, 13; table 1; figs. 2, 3C, 5B, 7.

v 2010 *Sculda* sp. – Haug et al., pp. 2, 6, 8; table 1; figs. 3A, B, 4E, 5C, D.

v 2011 *„Sculda” pusilla* – Haug & Haug, pp. 16–18; Abb. 3C, 4C.

v 2012b ?*Sculda pusilla* – Haug et al., fig. 7A, C.

v 2012b a single Mesozoic specimen – Haug et al., p. 11.

v 2015 ?*S. pusilla* – Haug et al., pp. 121, 131.

Derivation of the name: In honour of Laura Frattigiani, Laichingen. Her father Roger has a tremendous fossil collection, which he is opening for scientists since many years and which includes material for this study.

Holotype: SMNS 67592/1 (069, formerly collection Hermann Polz, Geisenheim).

Type locality: Blumenberg quarry near Eichstätt, west of Wegscheid (see Edlinger, 1964, his Abb. 20).

Type horizon: Altmühltal Group, Eichstätt Subformation (Lower Tithonian, Hybonotum Zone, Riedense Subzone) (Schweigert, 2007).

Paratypes: SMNS 67505 (017, collected by W. Ludwig, 1992), SMNS 67592/2 (070, formerly collection Hermann Polz, Geisenheim), SMNS 67593 (083, formerly collection Norbert Winkler, Stahnsdorf), SMNS 67633 (090, formerly collection Michael Fecke, Lippstadt).

Diagnosis: Stomatopod of moderate size. Body subcylindrical. Posterior feeding apparatus (maxillipeds 2–5) with first pair of raptorial appendages large, second pair of medium size, posterior two pairs being small. No dorsal surface ornamentation on shield or tergites. Pleopods with multi-annulated (outer ?) ramus. Uropods with lanceolate to paddle-shaped endo- and exopods. Endopods of uropods with serration along the median margin. Exopods of uropods with movable teeth along the lateral margin.

Remarks: Specimens now ascribed to this new species have been referred to as ?*Sculda pusilla* in Haug et al. (2010, and in subsequent publications referring to this, see above) as they lack the dorsal spine rows similar to the type specimen of “*Sculda pusilla*”. We now interpret “*Sculda pusilla*” as a nomen dubium (see also below).

### Remarks (differential diagnosis):

Shield: The shield of the representatives of *Sculda* (see Haug et al. (2010) for the problems of determining the number of different species) appears to be dorso-ventrally flattened, while that of the representatives of *Tyrannosculda* is more laterally flattened (subcylindrical). Gastric grooves are developed in both, but a cervical groove and anterior-posterior oriented ridges, which are well developed in *Sculda*, are missing in *Tyrannosculda*.

Raptorial appendages: The raptorial appendages of *Tyrannosculda* are differentiated as one large, one medium-sized and two small pairs of raptorial appendages, while in *Sculda* the size difference between the first and second pair of raptorial appendages appears to be (autapomorphically?) less well developed. More importantly, the propodi in *Sculda* are enlarged in width, while those of *Tyrannosculda* are simple ovate. Also opposing spines as developed in *Tyrannosculda* are not known to be present in *Sculda*.

Pleomere tergites: The pleomere tergites of *Sculda* bear a specific ornament of rows of backward-pointing teeth. Representatives of *Spinosculda* bear a pair of backward-pointing movable spines dorso-laterally on the last pleomere. In *Tyrannosculda* all pleomere tergites are simple smooth without any ornament and spines.

Pleopods: The pleopod morphology of *Tyrannosculda* differs significantly from that known from species of *Sculda*. The exact identity of endopod and exopod is difficult to evaluate due to the lateral preservation of the specimens, but we can make certain assumptions based on morphology of other fossil stomatopods (see also description). One of the two rami (the possible endopod) appears to be simple paddle-shaped with few (two or three) distal setae. The other ramus (the possible exopod) appears to be multi-annulated with four or five annuli. In *Sculda* both rami are paddle-shaped and bear significantly more setae along their margin, even in very small specimens.

Also, the uropods of *Tyrannosculda* differ significantly from those of *Sculda*. In particular, the spination is much more prominent in *Sculda* than in *Tyrannosculda*.

## Discussion

### Stepwise evolution of characters in mantis shrimps

*Tyrannosculda laurae* (Fig. 5A), treated by Haug et al. (2010) as ?*Sculda pusilla*, was not resolved in their analysis in relation to ?*Sculda pennata/spinosa* and Unipeltata sensu stricto (including the modern forms, Verunipeltata). Yet, this indicates that *T*. *laurae* is closer related to modern forms than all Carboniferous species, including species of the groups *Gorgonophontes* and *Tyrannophontes*.

With our new data it becomes furthermore clear that *T*. *laurae* and ?*S*. *pennata/spinosa* (Fig. 5B) differ significantly in their morphology, and that ?*S*. *pennata/spinosa* is more closely related to modern mantis shrimps than is *T. laurae*. In fact, *T*. *laurae* retains numerous plesiomorphic features. It differs from Carboniferous species (Figs. 5C–5D) through the acquisition of gastric grooves on the shield and the reduction of the thoracic tergites, at least of trunk segment three (further anterior ones unclear in Carboniferous species). These characters are shared with ?*S*. *pennata/spinosa* and Unipeltata sensu stricto. Another autapomorphy of the monophyletic group (?*S*. *pennata/spinosa* + Unipeltata sensu stricto) could be the presence of movable spines on the exopod of the uropod. The plesiomorphies shared with the Carboniferous representatives include the multi-annulated exopods on the pleopods, the shrimp-like pleon, and the triangular telson. In ?*S*. *pennata/spinosa*, as in modern forms, pleopod exopods are paddle-shaped, the pleon is more lobster-like in being dorso-ventrally flattened, and the telson is more square-shaped with a paired central tip instead of a single one.

### Reptantisation in the decapodan lineage

**Figure 6:**
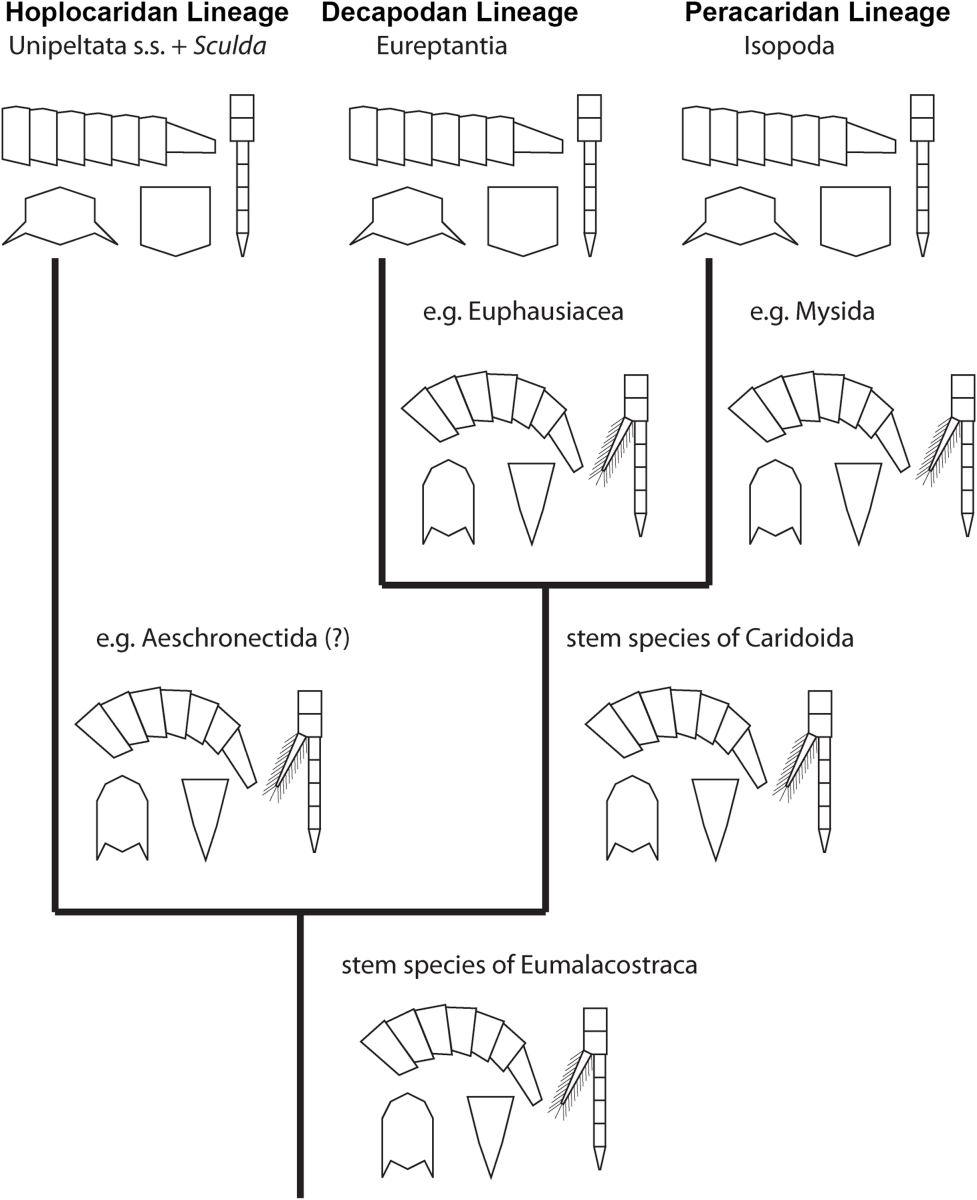
Convergent evolution of a reptantian morphotype in different eumalacostracan lineages. The drawings represent the character states in the adults at different points along the lineages. Explanation of drawings, clockwise starting lower left (two possible character states in brackets): pleon cross section (laterally flattened/dorso-ventrally flattened), pleon flexibility (limited ability to curl and straighten/large ability to curl and straighten), thoracopods (with exopods/without exopods), telson (triangular/rather square-shaped).

As mentioned in the introduction, the evolution from a shrimp-like morphotype to a more lobster-like morphotype occurs not only in the hoplocaridan lineage, but in the decapodan lineage as well (Fig. 6) (e.g., Bracken-Grissom et al., 2014). For a comparison of mantis shrimps with decapodan lobsters we have to assess the evolutionary pattern from the caridoidan shrimp morphotype to the decapodan lobster morphotype. If we start by comparing decapodans of the shrimp morphotype and of the lobster morphotype, different characters become apparent that make these two morphotypes so well differentiable. These characters are:

- The cross section of the body, especially the pleon. It is laterally flattened in the shrimp morphotype, but dorso-ventrally flattened in lobster morphotype.
- The ability to straighten and curl the pleon. In the shrimp morphotype the pleon is usually held in a rather bent position (note that it may often appear straight, but only because it is raised at the shield-pleon transition; the dorsal line is still clearly curved). In the lobster morphotype the pleon can be fully straightened, although many representatives hold the pleon partially folded most of the time. This ability appears to be (at least partly) coupled to the more flattened pleon segments in the lobster morphotype.
- The shape of the telson. It is relatively narrow and triangular in the shrimp morphotype, but usually broad and more or less square-shaped in the lobster morphotype (for exceptions, see below).
  If we look further down the lineage towards decapodan lobsters, more characters become apparent that differ between the lobster morphotype and the “classical” shrimp morphotype as seen in Euphausiacea or Mysida. The general morphology of these two groups are most likely relatively close to the ground pattern of Caridoida, with this to the ancestral shrimp morphotype. These forms differ from the lobster morphotype also in:
- the presence of exopods on the thoracopods, which are only found in larval stages of decapodans, but are absent in the majority of corresponding adults;
- the absence of a “sprawler” position of the posterior thoracopods, which is present in the benthic lobsters.

This comparison already shows that the lobster morphotype, which is recognised here on five specific characters, evolved stepwise from the shrimp morphotype (in the stricter sense). In fact, this demonstrates that the shrimp morphotype is not a distinct one, but several shrimps have already some lobster-like characters. At first, decapodan shrimps reduced the exopods on their thoracopods. Possibly, in a next step the “hanging” position of the thoracopods was changed to a sprawler one (possibly in the stem-species of Pleocyemata or Reptantia+Stenopodida, partly depending on the exact phylogeny of Decapoda; e.g. Scholtz & Richter, 1995; Tsang et al., 2008 and references therein). At the node of Reptantia the lobster-type pleon evolved with its specific cross section and the ability to straighten and curl it.

One type of lobster retained the plesiomorphic triangular telson; it is still present in representatives of Polychelida. Therefore, a sister group position of Polychelida to the remaining reptantian decapodans, Eureptantia, is a plausible explanation (e.g. Scholtz & Richter, 1995; Ahyong & O’Meally, 2004; a supposed position of Polychelida somewhere within a ‘lobster clade’ is therefore seen as unparsimonious, e.g. Tsang et al., 2008; Wolfe et al., 2019; reconstructions resolving polychelidans in such a position should discuss the retention of a shrimp-type telson in Polychelida). Eureptantia finally evolved the square-shaped telson, “finishing” the process of reptantisation.

The fully resolved order of the evolutionary steps from a shrimp-like to a lobster-like morphotype in Decapoda is reconstructed as: (1) loss of exopods on (at least some) thoracopods, (2) evolution of sprawler stance, (3) cross section of pleon dorso-ventrally flattened + straightening and curling of pleon, (4) evolution of a square-shaped telson. Even if we would accept the non-parsimonious idea that Polychelida is not the sistergroup to the remaining lobsters (as suggested by Tsang et al. (2008) or Wolfe et al. (2019)), the order would still be reconstructable as: (1) loss of exopods on (at least some) thoracopods, (2) evolution of sprawler stance, (3) cross section of pleon + straightening and curling of pleon + evolution of a square-shaped telson.

### Reptantisation and the evolution of the ‘mantis lobster’

The five novelties characterising the decapodan lobster type, better the eureptantian lobster type, can all also be found in modern mantis shrimps, Verunipeltata:

- the pleon is dorso-ventrally flattened;
- the pleon can be held straight (and curled, but slightly differently);
- the telson is more or less square-shaped and relatively broad;
- the exopods of the, at least anterior five, thoracopods are absent in the adults;
- the thoracopods (that are used for walking) are in a wide stance. Interestingly, this position in mantis shrimps is achieved in a different way than in decapodans. In eureptantian lobsters, such a position is achieved by sprawling due to a Z-shaped arrangement of the appendage elements. In modern mantis shrimps, the wide stance appears to be achieved by a very broad sternal region. While some eureptantians also have wider sternal regions (Scholtz & Richter, 1995; fig. 10B), the sprawler-type appendages allow to assume a wide stance also with a very narrow sternal region (Scholtz & Richter, 1995; fig. 10A).

While all modern mantis shrimps show all these five ‘lobster’ specialisations, the reconstruction of the stepwise evolution of characters (see above) allows also here to observe the evolution from a shrimp morphotype to a lobster morphotype. Within Hoplocarida, the supposed sister group to Stomatopoda is Aeschronectida (e.g., Schram, 1969; Jenner, Hof & Schram, 1998; although, admittedly, representatives of this group should be re-investigated). Representatives of Aeschronectida retain a general shrimp morphotype including exopods on the thoracopods. We have no observation of exopods on the raptorial appendages in any representative of Stomatopoda; loss of the exopods on (adult) thoracopods in consequence appears to be an apomorphy of Stomatopoda. Many early mantis shrimps retain their shrimp-like habitus, laterally flattened and curved pleon, triangular telson, hanging walking appendages (e.g. Schram, 1969, 2007; Jenner, Hof & Schram, 1998; Schöllmann, 2004). The transition of these characters to the lobster morphotype all take place in the stem species of ?*Sculda pennata/spinosa* + Unipeltata s. str.. It is unfortunate that we can achieve no further resolution of this evolutionary process; this indicates that we still miss certain split events.

Additionally, among early representatives of Stomatopoda another group appears to have evolved a partly lobster-like morphotype. The 300 million-year-old fossil species of *Perimecturus* are quite broad and possess a dorso-ventrally flattened pleon, which appears to have been straight (e.g. Jenner, Hof & Schram, 1998). Whether the thoracic appendages in these species allow a wide stance or not is unclear; the telson retains its triangular shape.

As a consequence, we can still call Stomatopoda ‘mantis shrimps’ as the stem species of this monophyletic group was indeed shrimp-like. Yet, all modern forms (Verunipeltata) clearly possess the morphology of a ‘mantis lobster’. We therefore suggest to refer to the monophyletic group consisting of ?*Sculda pennata/spinosa* + Unipeltata s. str. as Stomatoreptantia or mantis lobsters.

Hence, the exact order of evolutionary steps from a shrimp-like to a lobster-like morphotype in Hoplocarida cannot be fully resolved. Yet, the loss of exopods on (at least some) thoracopods apparently was the first evolutionary step also in Hoplocarida as has been the case in Decapoda. At least when considering the lineage towards *Perimecturus*, the dorso-ventral flattening of the pleon evolved afterwards, while a square-shaped telson was never evolved in this lineage. With this, there are certain similarities to the evolution in Decapoda. For the lineage towards Stomatoreptantia, the exact order of evolutionary steps after the loss of exopods cannot be resolved.

### Another possible example: Isopoda

Isopodans, more exactly representatives of certain ingroups, also appear to have evolved towards a lobster morphotype. Isopoda is an ingroup of Peracarida, which plesiomorphically also possesses a shrimp morphotype (as exemplified by Mysida and Lophogastrida). Representatives of Isopoda lack the exopods of the thoracopods (e.g. Richter & Scholtz, 2001). This seems to be therefore a ground pattern feature of the group. Early representatives of Isopoda (Phreatoicidea) retain a higher body cross section, the sister group (= the remaining representatives of Isopoda) appears to be characterised by a flattened body. While all representatives of Isopoda have a kind of a sprawler-type stance, it remains unclear when this evolved as there are sprawler-type stances in Tanaidacea and also Amphipoda (which both have lost exopods on at least some of the thoracopods). Yet, here the situation is complicated by the unusual behaviour coupled to this morphology, which is not lobster-like. Gammaridean amphipodans, for example, push themselves forward with these appendages while lying on the side of their clearly shrimp-like body, so the thoracopods are not used for walking on the ground as it would be the case in a lobster-like mode. The exact node at which a sprawler-type stance evolved within Peracarida, or even whether it evolved several times independently, remains therefore currently unknown.

Finally, within Isopoda some representatives changed the telson shape from triangular to more square-shaped (e.g. *Bathynomus*) and possess a dorso-ventrally flattened pleon which can be fully straightened (e.g. Serolidae). This results in a rather lobster-like morphology in certain isopodans.

Hence, also here the exact order of character evolution from a shrimp-like to a lobster-like morphotype is not possible, yet it can be resolved to the following pattern: • loss of exopods on at least some of the thoracopods + sprawler-type stance, • dorso-ventral flattening of the pleon, • evolution of a square-shaped telson.

### Evolving a lobster morphotype

Based on observation of different evolutionary lineages we can infer a certain similarity in the pattern of character evolution from a shrimp morphotype to a lobster morphotype. The first step appears always to be the loss of the exopods on (some of) the thoracopods in the adults (Decapoda, Stomatopoda, possibly within Peracarida). As a second step, (some of) the thoracopods evolve the wide stance (in Decapoda, unclear in Stomatopoda, partly unclear in Peracarida). As a third step, the dorso-ventrally flattened pleon evolves (at least in Decapoda and Peracarida), which appears to be coupled to the ability to hold it straight. Lastly, in some cases a square-shaped telson evolves. Although this cannot be fully resolved for all lineages, the observed patterns do not contradict this order. Based on this partly re-occurring pattern, there appear to be some intrinsic limiting factors in which order of evolutionary events a lobster morphotype evolves.

How does it look for extrinsic factors? Loss of exopods could be seen as being coupled to a change from nektic or nekto-benthic to benthic life style as it occurs also during ontogeny in different decapodans (e.g. Haug, 2020). Yet, early decapodans are commonly called ‘Natantia’, swimmers. They swim by beating their pleopods as do modern mantis lobsters. Thus, no direct coupling between loss of exopods and change to benthic life style can be inferred. Also a dorso-ventrally flattened pleon does not appear to be necessarily coupled to a change to a benthic habitat, as demonstrated by numerous benthic groups retaining the shrimp-type pleon, such as different carideans and stenopodideans. A functional coupling of the telson shape is also not apparent. What seems to be directly coupled to a benthic life style is a wide stance of the thoracopods.

In conclusion, evolving a lobster morphotype, i.e. the process of reptantisation, appears to have one extrinsic factor, the change to benthic life style being coupled to a wide stance. Other morphological aspects of the evolution of a lobster morphotype appear to follow an intrinsic order. This order should be caused by functional constraints that are currently not apparent and should be investigated in future studies.

### Life style of mantis shrimps

*Tyrannosculda laurae* appears quite similar to Carboniferous stomatopodans, such as species of *Gorgonophontes* and *Tyrannophontes*. All these species have a large shrimp-type pleon and tail fan. Their ‘walking’ appendages, i.e. the posterior thoracopods, as far as known, appear rather short, not allowing a wide stance (e.g. Schram, 1969; Jenner, Hof & Schram, 1998).

While shrimp-type pleons can occur in species with benthic life habits (see caridean shrimps), these usually possess thoracopods that allow for a wide stance. Therefore, it seems unlikely that these early mantis shrimps possessed a benthic life style. Also other aspects of their morphology do not indicate such a life style. The tergites of the segments of the raptorial appendages of modern mantis lobsters, but also of extinct species like ?*Sculda pennata/spinosa*, are reduced in anterior-posterior axis or entirely unsclerotised to facilitate an uplifting of the anterior body. This appears to be necessary to allow a prey capture movement while standing on the ground. In Carboniferous species, the tergites are much more prominent, well sclerotised, and longer in anterior-posterior axis (e.g. Schram, 1969; Jenner, Hof & Schram, 1998). In *T*. *laurae*, these tergites appear relatively small. Yet, some of the segments that appear entirely unsclerotised in modern forms are still present as sclerites in *T*. *laurae*. Furthermore, the (ventro-)laterally extending shield would not have allowed an uplifting of the anterior body in a comparable way to mantis lobsters. We, therefore, assume that early mantis shrimps indeed hunted prey while swimming, possibly as necto-benthic predators, grabbing prey from above. In modern forms, mainly the larvae hunt while swimming (e.g. Pyne, 1972). As Haug et al. (2010) already pointed out, for a better understanding of the functional morphology of extinct mantis shrimps we need a better understanding of the functional morphology of modern stomatopodan larvae.

### Another survivor

Haug et al. (2012c) discussed the survival of specific morphotypes over geological time. They discussed cases in which supposed typical Cambrian morphotypes surprisingly survived into the Devonian or even Carboniferous (other examples are the great-appendage arthropodan *Schinderhannes bartelsi* (Kühl, Briggs & Rust, 2009), or different organisms from the Moroccan Fezouata Formation (Van Roy, Briggs & Gaines, 2015)). An example of such a morphotype survival in younger geological times was recently described by Godunko, Staniczek & Bechly (2011). Here insect nymphs of a morphotype that could be termed ‘typical’ for the Carboniferous were described from Triassic and Cretaceous deposits. *Tyrannosculda laurae* represents a comparable example as major aspects of a Carboniferous morphotype survived into the Jurassic. As in the example of Haug et al. (2012c) this ‘ancient’ morphotype interestingly co-occurs with closely related species with a relatively modern morphotype.

As both morphoytypes fulfilled different ecological functions, they were not in direct competition. Yet, specialised giant larvae of modern mantis lobsters, as discussed above, appear to have fulfilled a relatively similar ecological function as adults of *T*. *laurae*. As such specialised larvae also seem to appear in the Jurassic (Haug, Haug & Ehrlich, 2008; Haug, Wiethase & Haug, 2015), these could have been directly competing with swimming adult mantis shrimps. Finally, the niche differentiation between early stages (nektic to planktic) and later ones (benthic) might have been the more successful life history strategy as it avoids competition for resources between different life stages of the same population.

## Conclusions

The here described new mantis shrimp *Tyrannosculda laurae* exhibits a morphology bridging the gap between shrimp-like Carboniferous forms and lobster-like younger forms. Furthermore, it provides hints to the order in which the evolutionary steps towards the lobster-like morphology occurred in Stomatopoda. Similar evolutionary steps appear to have occurred also in other lineages within Malacostraca, but the exact order of all steps could not be resolved yet. Future fossil findings may provide additional clues on this aspect.

## Additional Information and Declarations

### Data Availability

The following information was supplied regarding data availability: Specimens used in this study come from the collections of the Staatliches Museum für Naturkunde Stuttgart with repository numbers SMNS 67505, SMNS 67592/1, SMNS 67592/2, SMNS 67593, and SMNS 67633. The presented data can be found in Figs. 1–4.

### New Species Registration

The following information was supplied regarding the registration of a newly described species: Publication LSID: urn:lsid:zoobank.org:pub:66FB761B-8C3E-4B57-8E0C-1055B2E8BD73. Tyrannosculda n. gen. LSID: urn:lsid:zoobank.org:act:912A8E58-2C1E-4107-A40A-E693CBB121F7. Tyrannosculda laurae n. sp. LSID: urn:lsid:zoobank.org:act:27B4B4E9-C598-4140-B34C-267EBAD68F6B. Stomatoreptantia (not governed by ICZN).
